# Crystallization Behavior and Mechanical Properties of Poly(ε-caprolactone) Reinforced with Barium Sulfate Submicron Particles

**DOI:** 10.3390/ma14092368

**Published:** 2021-05-02

**Authors:** Hegoi Amestoy, Paul Diego, Emilio Meaurio, Jone Muñoz, Jose-Ramon Sarasua

**Affiliations:** Department of Mining-Metallurgy Engineering and Materials Science, POLYMAT, Faculty of Engineering in Bilbao, University of the Basque Country (UPV/EHU), Plaza Torres Quevedo 1, 48013 Bilbao, Spain; hegoiamestoy@gmail.com (H.A.); paul.diego.martin@gmail.com (P.D.); emiliano.meaurio@ehu.eus (E.M.)

**Keywords:** poly(ε-caprolactone), crystallization kinetics, radiopacity, barium sulfate

## Abstract

Poly(ε-caprolactone) (PCL) was mixed with submicron particles of barium sulfate to obtain biodegradable radiopaque composites. X-ray images comparing with aluminum samples show that 15 wt.% barium sulfate (BaSO_4_) is sufficient to present radiopacity. Thermal studies by differential scanning calorimetry (DSC) show a statistically significant increase in PCL degree of crystallinity from 46% to 52% for 25 wt.% BaSO_4_. Non-isothermal crystallization tests were performed at different cooling rates to evaluate crystallization kinetics. The nucleation effect of BaSO_4_ was found to change the morphology and quantity of the primary crystals of PCL, which was also corroborated by the use of a polarized light optical microscope (PLOM). These results fit well with Avrami–Ozawa–Jeziorny model and show a secondary crystallization that contributes to an increase in crystal fraction with internal structure reorganization. The addition of barium sulfate particles in composite formulations with PCL improves stiffness but not strength for all compositions due to possible cavitation effects induced by debonding of reinforcement interphase.

## 1. Introduction

In recent years, there has been an increasing interest in biodegradable polymers for biomedical applications as temporary surgical implants and templates for tissue engineering. Polyesters based on polylactides and polylactones present tremendous interest in this field because of their biocompatibility, tunable mechanical properties and biodegradability [1,2,3,4,5,6]. Among these polymers, poly(ε-caprolactone) (PCL) stands out, as it widely used in tissue engineering and drug delivery platforms [7,8,9,10,11,12].

Despite its success as implantable material, in comparison to metals, its low radiopacity makes it difficult to detect by X-ray imaging techniques. Hence, to overcome this difficulty, the use of composites with high radiopaque fillers is being investigated. Barium sulfate (BaSO_4_) [13,14,15], ferrous oxide (Fe_3_O_4_) [16] and bismuth oxide (Bi_2_O_3_) [17,18] have been previously reported as radiopaque composite fillers, and BaSO_4_ is currently the most commonly used in medical applications. Barium sulfate (BaSO_4_) is a standard in medical applications, approved by the FDA and used for X-ray assisted implantation to control the position of polymer implants and drug delivery systems during biodegradation [9,19,20].

The incorporation of BaSO_4_ has been reported to be an alternative to improve the toughness of, for example, polylactides [21,22]. However, its use also might negatively affect other mechanical properties depending on the particle content. For instance, barium sulfate as an inorganic reinforcement has been already reported to be used with various polymer matrices such as polyethylene, polypropylene and poly(lactic acid); performance was reported to be reduced at large filler contents [23,24,25,26]. The fail in mechanical properties is often explained in terms of poor compatibility of the matrix–reinforcement interface [27]. In the particular case of PCL, a number of authors have demonstrated poor compatibility with other fillers [28,29,30]. These two facts, the relative content of the particle and the matrix–reinforcement interface, play an important role in the correct use of PCL-based devices. On the one hand, to obtain sufficient radiopacity, weight percentages of about 25% are necessary for commonly used BaSO_4_ particles in medical applications. On the other hand, the particle content that ensures sufficient radiopacity must preserve or improve the mechanical properties of the base material. Therefore, an equilibrium must be found between these two variables.

Moreover, incorporating inorganic particles in a crystallizable polymer matrix affects the crystallization kinetics, crystal morphology and final degree of crystallinity of the base polymer [22,31]. Therefore, in this work, a complete study of crystallization of PCL in presence of BaSO_4_ particles is presented. PCL at human body conditions (≈37 °C) is above its glass transition temperature (*T*_g_ ≈ −60 °C). At these conditions, mechanical properties of PCL are soft and strongly related to degree of crystallinity. In fact, previous research has shown the important effect of fillers on polymer crystallinity depending on the composition, content, size and morphology of the reinforcement [29,30,32]

There is growing evidence in the literature that recognizes the importance of the development of biodegradable radiopaque polymeric systems for being used as medical devices. Studies over the past two decades have provided important information on the physical–mechanical properties of PCL. However, to date, the relationship between crystallization behavior, mechanical properties and radiopacity upon BaSO_4_ particle addition has not yet been extensively studied. Therefore, the aim of this research work was to study the role of BaSO_4_ in the crystallization behavior, mechanical properties in terms of the particle–matrix interface and radiopacity, establishing the minimum BaSO_4_ content needed to preserve the mechanical performance. 

## 2. Materials and Methods

Poly(ε-caprolactone) (PCL) CAPA6500, with an average molecular weight (*M*_w_) of 147.6 KDa and a dispersity index (*D*) of 1.49 was provided by Solvay (Perstorp, Sweden). Barium sulfate (BaSO_4_) was provided by Fluka Analytical (Sigma Aldrich). Particle size was determined by dynamic light scattering (DLS) on a Zetasizer ZS90 ZEN3690 (Malvern Instrument, UK), showing an average diameter of 577 ± 125 nm.

### 2.1. Sample Preparation

PCL and BaSO_4_ composites were prepared by melt mixing in an MC5 conical twin-screw mixer (Xplore, The Netherlands) at 150 °C and 150 rpm for 30 min. Compositions with 5, 15, 25 and 35 wt.% BaSO_4_ were prepared (see name codes in Table 1). Sheets of 1 mm thickness of neat PCL and composites were prepared from pellets by compression molding in a Dr. Collin P200E hot press (Collin, Germany) at 150 °C and 25 MPa. 

The amount of barium sulfate present in samples after processing was measured by thermogravimetric analysis (TGA) in a TGA model Q50 (TA instruments). Samples of 10–15 mg were heated from room temperature to 500 °C at a rate of 10 °C min^−1^ under a nitrogen atmosphere. Three different samples (*n* = 3) were used to determine the content of BaSO_4_ in the samples. A slide difference was found between the actual and real content of the particle (see Table 1).

### 2.2. Non-Isothermal Crystallization Kinetics 

Before the non-isothermal crystallization treatments, the thermal history of the samples was erased by increasing the temperature to 100 °C, and then non-isothermal crystallization treatments were carried out from melt by cooling the samples at cooling rates of 1, 5 and 10 °C min^−1^. Thermal properties of the samples were measured in a subsequent differential scanning calorimeter (DSC) run by heating from –85 to 100 °C at a rate of 20 °C min^−1^ in a nitrogen atmosphere. The DSC employed was a Q80 differential scanning calorimeter (DSC) (TA instruments) calibrated with pure indium and sapphires. In all cases, three different samples (*n* = 3) weighting 7 ± 1 mg were used. Cooling cycles were monitored. The melting temperature (*T*_m_) was determined from the endothermic peak position, and the melting enthalpy (Δ*H*_m_) was obtained by calculating the area under the melting peak.

### 2.3. Crystal Morphology

Spherulitic morphology of samples was observed with a LEICA DM LM polarized light optical microscope (PLOM) (LEICA, Spain). Samples were dissolved in dichloromethane to obtain films by solvent casting on a microscope glass slide. Non-isothermal crystallization treatments were conducted by a heat controlling Mettler FP90 hot stage (Mettler Toledo, Spain) at the conditions described previously. 

### 2.4. Mechanical Testing

Tensile testing was conducted in an Instron 5565 universal testing machine according to ISO 37-2 standard. Samples of 4 mm width were cut from the molded sheets, and at least five samples (*n* = 5) were employed for mechanical properties measurements. Testing was performed at a crosshead speed of 10 mm min^−1^. Young’s modulus (*E*) with a linear fitting at initial 2% of strain, yield strength (σ_y_), stress at break (σ_b_) and strain at break (ε_b_) were determined.

### 2.5. X-ray Images

Radiographs of samples were taken using an X-radiographic standard clinical machine X-ray IRIS 70 (Trophy, UK). As a reference, a 1 mm aluminum plate was employed. All the samples including the reference and neat PCL were tested using the same intensity for comparative purposes. Exposure time was 0.16 s.

## 3. Results

### 3.1. Crystallization Behavior and Crystal Morphology

**Figure 1:**
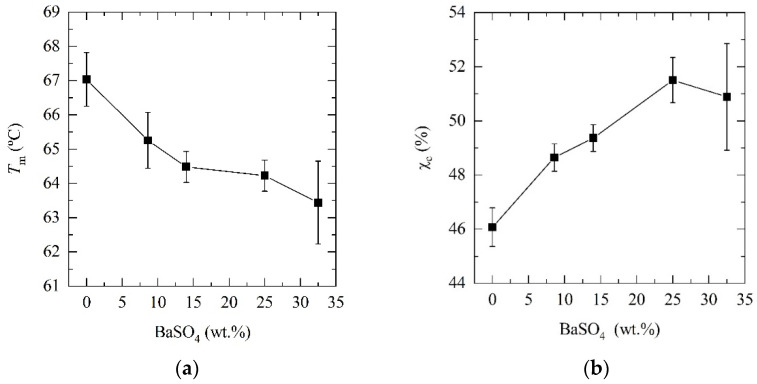
(**a**) Melting temperature and (**b**) degree of crystallinity of neat PCL and its barium sulfate (BaSO_4_) composites.

The incorporation of barium sulfate (BaSO_4_) decreased the melting temperature and increased the degree of crystallinity of poly(ε-caprolactone) (PCL), as revealed by diferential scanning calorimetry (DSC) (see Figure 1 and Table 2). The melting temperature monotonously decreased from 67 °C for neat PCL to 63.4 °C when incorporating 35 wt.% BaSO_4_, suggesting smaller and less perfect crystalline structures. The degree of crystallinity (*X*_c_) of PCL and its composites was calculated according to Equation (1), where Δ*H*_m_ is the melting enthalpy, Δ*H*_m_° is the melting enthalpy of 100 % crystalline PCL taken as 139 J g^−1^ [33] and *X*_BaSO__4_ is the real barium sulfate fraction in samples obtained by thermogravimetric analysis (TGA) (see Table 1).

(1) $$X_{c}\left(\%\right)=\frac{\Delta H_{m}}{\Delta H_{m}^{{}^{\circ}}}\times \left(\frac{1}{{1-X}_{\mathrm{BaSO}4}}\right)$$

The degree of crystallinity increased from 46% to 52%, which is statistically significant for compositions of 25 and 35 wt.% (*p* = 0.03 and *p* = 0.007).

**Figure 2:**
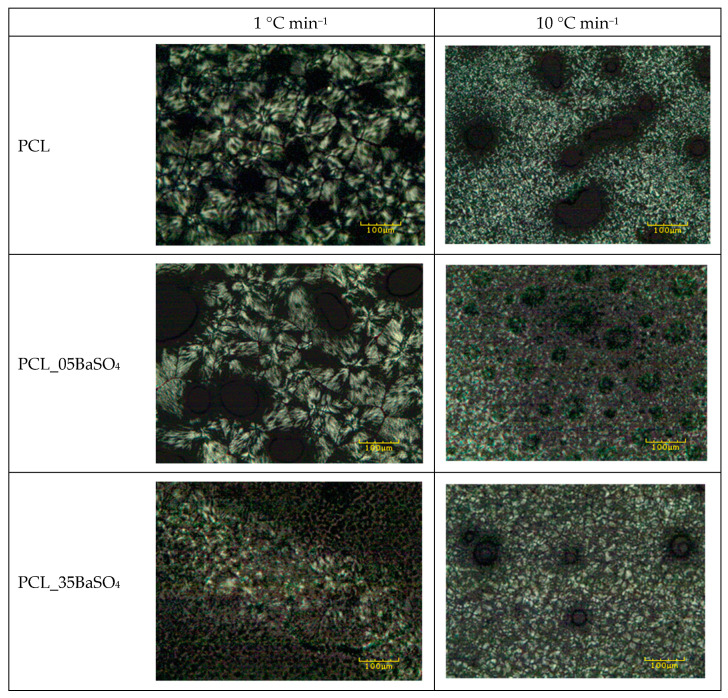
Crystal morphology for neat PCL and its composites having 5 and 35 wt.% BaSO_4_ after non-isothermal crystallization at cooling rates of (left column) 1 °C min^−1^ and (right column) 10 °C min^−1^. Black holes in micrographs correspond to air bubbles formed during in situ crystallization treatments.

PCL and its BaSO_4_ composites were subjected to non-isothermal crystallization treatments in situ in a polarized light optical microscope (PLOM). The resultant crystal morphology after samples were cooled from melt at cooling rates of 1, 5 and 10 °C min^−1^ were studied and compared. As expected, smaller spherulites were observed in samples cooled at higher cooling rates than in their counterparts due to molecules having less time for diffusion. In Figure 2, images taken at cooling rates of 1 and 10 °C min^−1^ are compared for neat PCL and PCL containing 5 and 35 wt.% BaSO_4_. Regardless of the cooling rate, no significant differences were found in crystal size between composites having low particle content, 5 wt.%, and neat PCL. However, the incorporation of higher particle contents, 35 wt.%, did have an influence on crystal size. For example, PCL shows a spherulitic structure with an average size of 100 µm and clear grain boundaries when cooled at 1 °C min^−1^, whereas the composite having 35 wt.% BaSO_4_ shows an average size of 5 µm. When comparing the crystal morphology of PCL at different cooling rates, the result reveals a highly distorted and more planar spherulitic structure at higher cooling rates. However, when BaSO_4_ particles are incorporated, this spherulitic morphology, though distorted as in neat PCL, becomes rounder.

### 3.2. Non-Isothermal Crystallization and Kinetics Analysis

**Figure 3:**
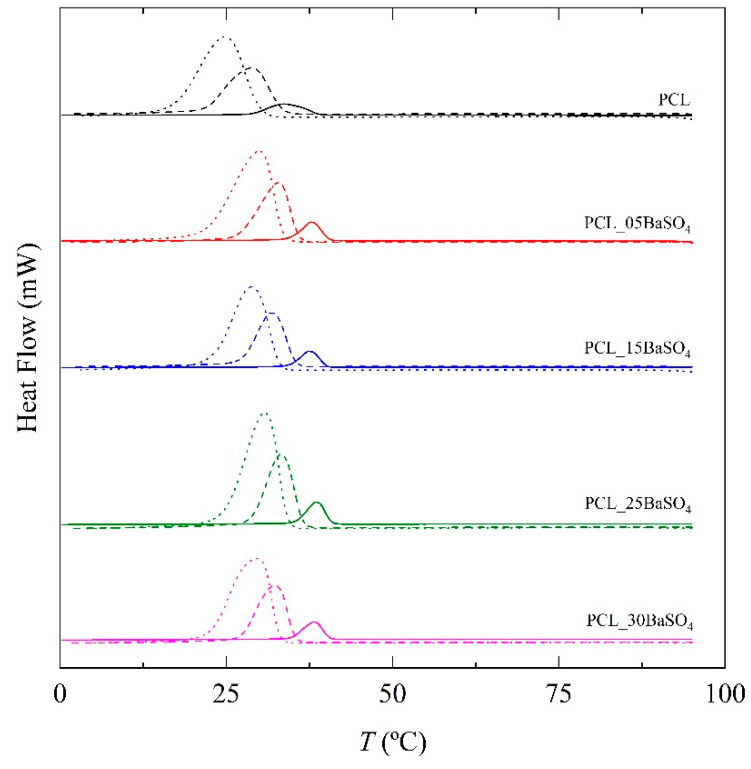
Cooling curves obtained by differential scanning calorimetry (DSC) for neat PCL and its BaSO_4_ composites at different cooling rates. Solid line: 1 °C min^−1^; dashed line: 5 °C min^−1^; dotted line: 10 °C min^−1^.

**Figure 4:**
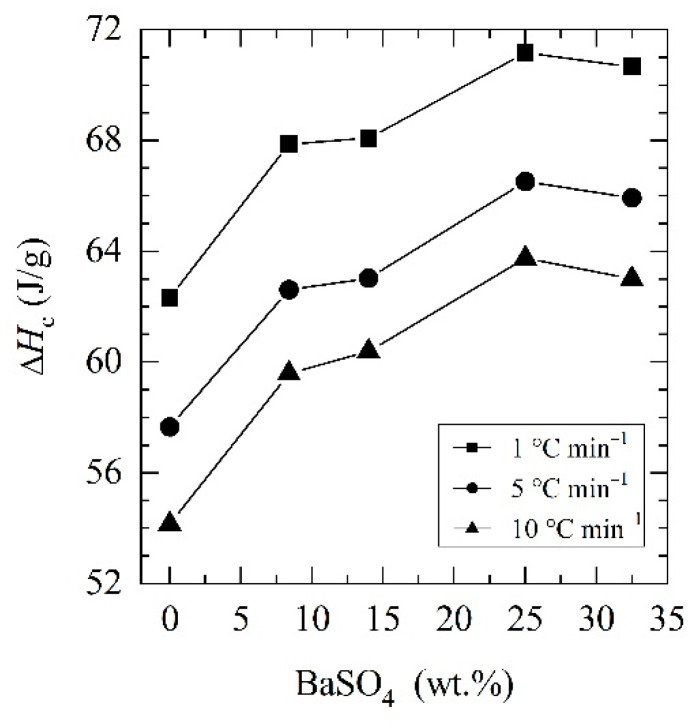
The increase in crystallization enthalpies of PCL and its BaSO_4_ composites obtained at (■) 1 °C min^−1^, (●) 5 °C min^−1^ and (▲) 10 °C min^−1^ cooling rates from melt.

To analyze in more detail the non-isothermal crystallization, cooling curves from DSC were analyzed for PCL and its BaSO_4_ composite cooled at cooling rates of 1, 5 and 10 °C min^−1^ (see Figure 3). A crystallization peak is observed between 30 and 40 °C for all compositions, yet in composites, this is observed at higher temperatures, suggesting heterogeneous nucleation induced by BaSO_4_ particles. At higher cooling rates, the crystallization peaks translate to lower temperatures for neat PCL and composite samples (see Table 3). Moreover, the crystallization degree during cooling increases with BaSO_4_ content as deduced from the increase in the crystallization enthalpy (see Figure 4). Therefore, the presence of the inorganic particles in PCL not only provides nucleation sites and accelerates crystallization kinetics but also leads to a higher degree of crystallinity. Furthermore, lower cooling rates favor both nucleation and the final degree of crystallinity in neat PCL and its composites. The crystallization peak temperatures and crystallization enthalpy for each composition at the selected different cooling rates are summarized in Table 3.

**Figure 5:**
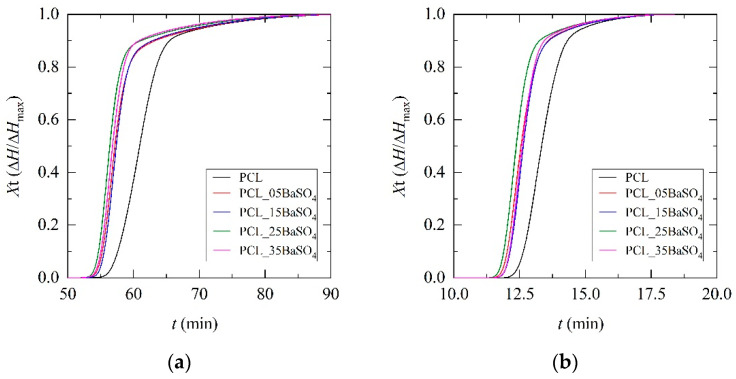

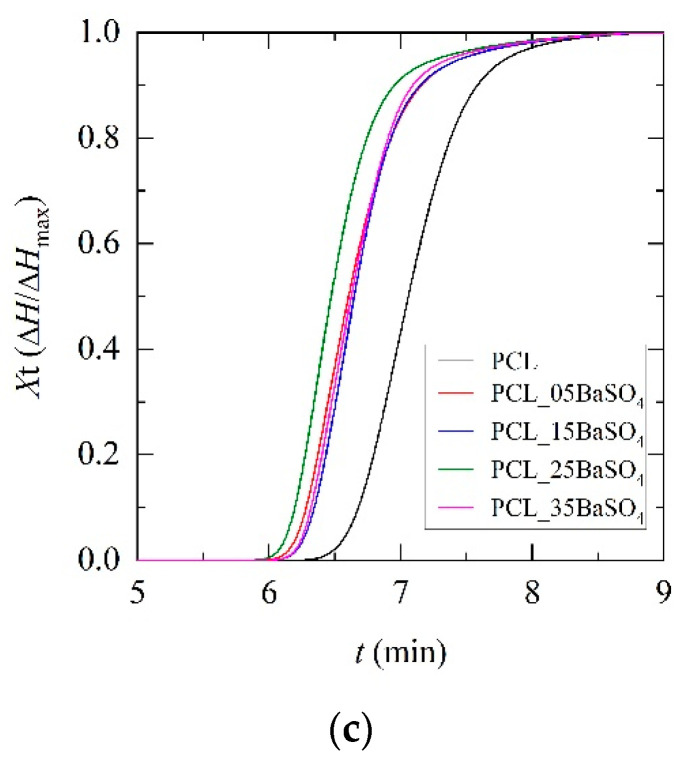
Degree of crystallinity versus time of PCL and its BaSO_4_ composites at different cooling rates: (**a**) 1 °C min^−1^; (**b**) 5 °C min^−1^; (**c**) 10 °C min^−1^.

The evolution of the degree of crystallinity with time is shown in Figure 5 for the different cooling rates. Crystallization kinetics was studied by analyzing the degree of crystallinity as a function of temperature according to Equation (2), where *T*_0_ and *T*_∞_ are initial and final temperatures of the crystallization process and d*H*_c_ is the crystallization enthalpy at temperature difference dT and at temperature *T*. The crystallization time during the process was assessed according to Equation (3), where T_c_ is the crystallization temperature at crystallization time *t* and *Φ* is the cooling rate.

(2) $$X\left(T\right)=\int_{T_{0}}^{T}\left(\frac{dH_{c}}{dT}\right)dT/\int_{T_{0}}^{T_{\infty }}\left(\frac{dH_{c}}{dT}\right)dT,$$

(3) t=(Tc−T)/Φ.

In accordance with DSC results, the evolution of the degree of crystallinity over time indicates the nucleating effect of BaSO_4_ particles advancing the onset of crystallization in regard to PCL. Moreover, the results revealed that crystallization might be accelerated by the presence of BaSO_4_. This was further assessed by employing crystallization models. 

For crystallization kinetic analysis, the Avrami-Ozawa-Jeziorny (A-O-J) model was employed. Polymer crystallization kinetics can be described by Avrami’s model for phase changes at isothermal conditions [34] by Equation (4), where 1 – *X*_t_ is the non-crystallized volume fraction, “*n*” is a parameter that defines the type of crystal growth and *Z*_t_ is a combined factor of nucleation rate and crystal growth rate. In non-isothermal conditions, the Ozawa model [35] can be employed by following Equation (5). Note that both equations are quite similar, although the *Z*_t_ and “*n*” parameters do not have the same physical meaning because the temperature varies constantly in non-isothermal crystallization. For our study, the Ozawa model could not be employed as some bibliographic results show that this model does not satisfactorily fit the PCL crystallization process [36,37]. Therefore, the change to *Z*_t_ proposed by Jeziorny [38] was applied for Avrami´s model being used in non-isothermal conditions. Equation (6) describes the change proposed by Jeziorny in order to unify Avrami´s and Ozawa’s models for non-isothermal conditions. In this equation, Φ is the cooling rate and *Z*_c_ a factor obtained from non-isothermal conditions with the equivalent meaning of *Z*_t_ in the Avrami model. In order to obtain “*n*” and *Z*_c_ factors, a linear fitting must be applied for log(–ln(1 – *X*_t_)) versus log(t) curves.

(4) $$1-X_{t}=e^{-Z_{t}\cdot t^{n}},$$

(5) $$1-X_{t}=e^{-Z_{t}\cdot \Phi^{m}},$$

(6) $$\log\left(Z_{c}\right)=\frac{\log\left(Z_{t}\right)}{\Phi }.$$

**Figure 6:**
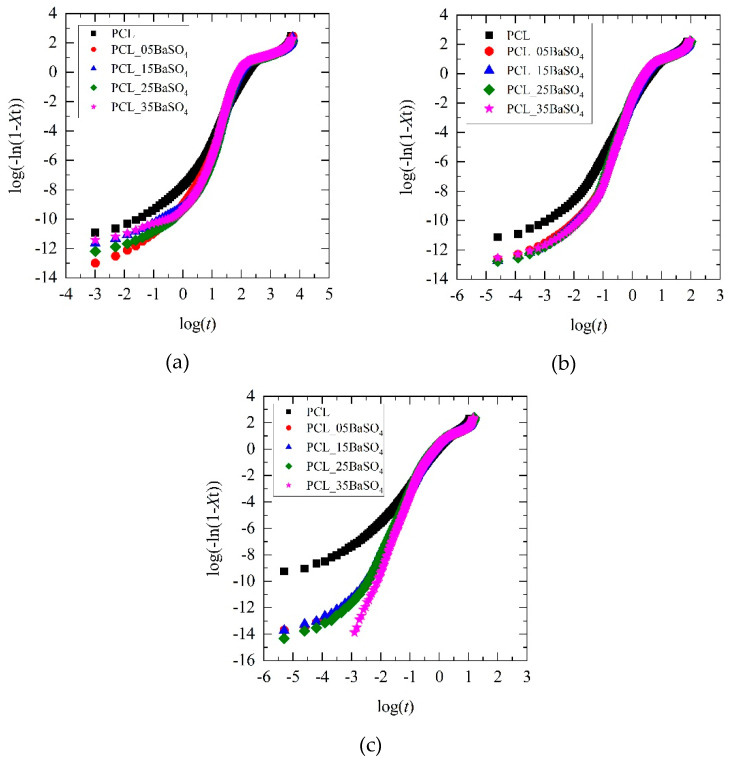
Plots showing log(–ln(1 – *X*_t_) versus log(*t*) of PCL and its BaSO_4_ composites at different cooling rates, used to adjust the Avrami-Ozawa-Jeziorny model: (**a**) 1 °C min^−1^; (**b**) 5 °C min^−1^; (**c**) 10 °C min^−1^.

Figure 6 shows the double logarithmic curves of PCL and its composites fitting the crystallization model proposed. The experimental data do not fit in a simple linear manner and show in all cases a more complex crystallization behavior. This finding is consistent with reported bibliographic results for PCL, suggesting the existence of a secondary crystallization process [36,37,39]. This secondary crystallization is common in other polymers with similar thermal properties, such as HDPE, when the working conditions are high above the glass transition temperature [40]. Therefore, in this work, a double linear behavior was considered for data fitting, obtaining positive results (*r* ≥ 0.9). 

**Figure 7:**
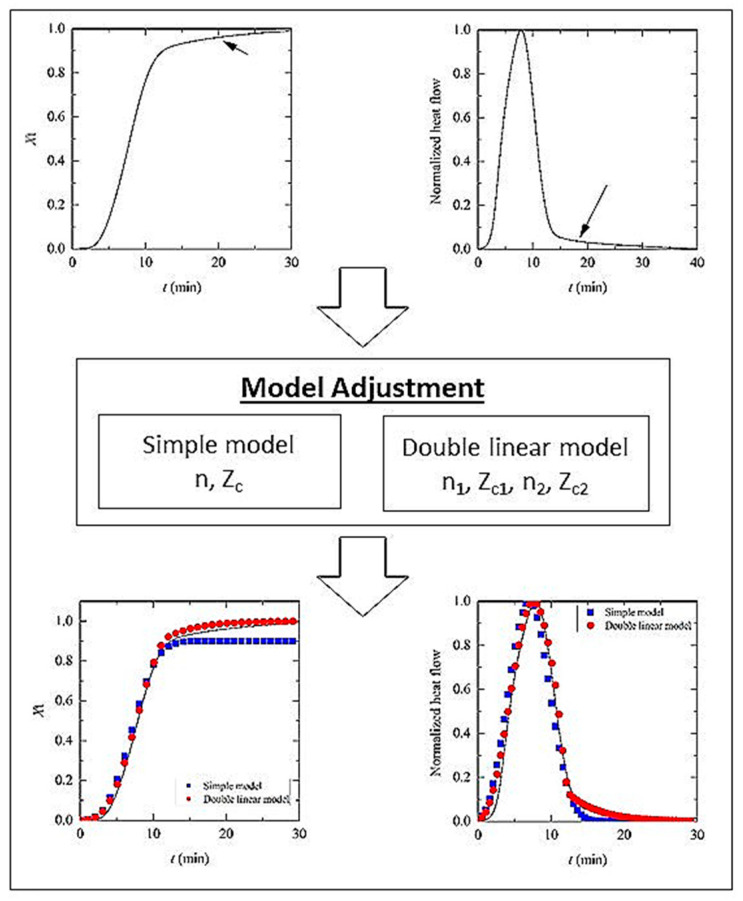
Example of simple (blue) and double linear (red) fitting models of experimental results of crystallization kinetics for PCL and its composites. Black arrows indicate the double-fitting area corresponding to the secondary crystallization. Data shown correspond to neat PCL at 1 °C min^−1^ cooling rate

Figure 7 shows an example of the simple and double linear model adjustments of crystallization kinetics of PCL and its BaSO_4_ composites. The values of the double fitting model parameters (*n*_i_ and *Z*_ci_) are summarized in Table 4. 

For the first linear fitting, n_1_ is always higher for composite samples and is close to the theoretical factor of 4corresponding to a three-dimensional crystallization according to the model. In contrast, neat PCL presents a factor closer to 3, which slightly decreases when the cooling rate is increased. The values for composite samples do not change with cooling rate and are not dependent on BaSO_4_ quantity. For the same sample, *Z*_c1_ rises abruptly with the cooling rate due to the usual effect of nucleation enhancement by fast cooling. The increase in particle content shows an incremental effect on this factor at high cooling rates, 10 °C min^−1^, whereas for low cooling rates, 1 °C min^−1^, all samples have similar behavior. 

The kinetics study according to this model shows that nucleation of PCL crystals is led by the presence of BaSO_4_ particles in a primary crystallization process to form spherulitic morphology. In fact, PLOM images showed that for low cooling rates, nucleation and growth of crystals in PCL were favored. The presence of BaSO_4_ leads to heterogeneous nucleation points at the interfaces starting the crystallization process earlier than in neat PCL, with an increase in the degree of crystallinity also observed in previous results. When the cooling rate is increased, however, the nucleating effect becomes more relevant because the effect of particles on the crystallization of PCL is enhanced. 

Secondary crystallization is also important; up to 40% of total crystallization has been attributed to it [41]. Three reasons might be proposed to explain this: (1) new lamellae are formed in the amorphous phase, (2) lamellae become thicker or (3) new lamellae grow at particle interfaces. This secondary crystallization just after primary crystallization (identified with a black arrow in Figure 7) is observable in the DSC normalized cooling curves (Figure 3) and crystallinity versus time plots (Figure 5). An inclination that can be attributed to secondary crystallization can be observed. Irrespective of BaSO_4_ content, the value of the n_2_ factor is low, between 1 and 2, suggesting a process more related to molecular reorganization than crystal growth. For the same sample, this factor increases with cooling rate, leading to distorted spherulitic crystals in accordance with the results obtained from PLOM analysis. 

### 3.3. Mechanical Properties

**Figure 8:**
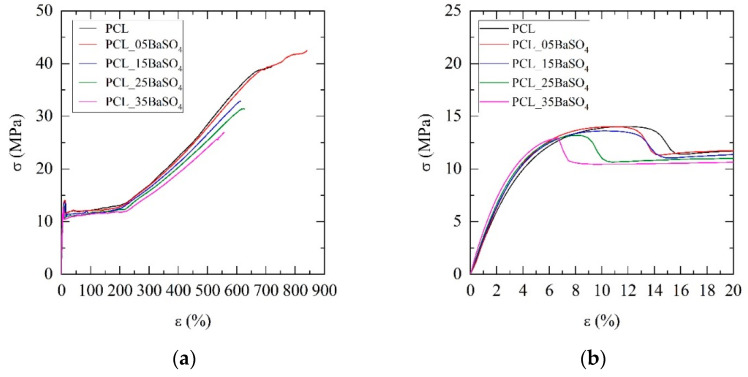

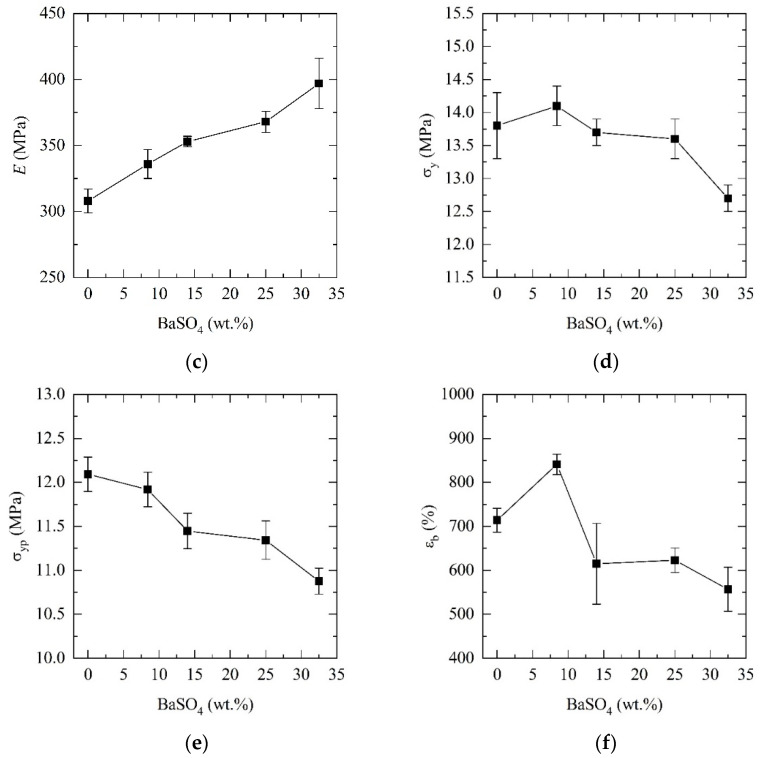
Mechanical behavior of PCL and its BaSO_4_ composites: (**a**) stress-strain curves; (**b**) close-up of the plastification peak at the yield point. Evolution of the mechanical properties: (**c**) Young´s modulus; (**d**) yield stress; (**e**) yield plateau; (**f**) elongation at break.

The stiffness of PCL was significantly increased with the increase in barium sulfate content in composites, with values of Young’s modulus ranging from 308 MPa for non-reinforced PCL to 397 MPa for its 35 wt.% BaSO_4_ composite counterpart. This effect on modulus was statistically significant starting from the 15 wt.% composite (*p* = 0.018) (see Figure 8c and Table 5).

The yield strength remained constant irrespective of the presence of BaSO_4_ content until 35 wt.% BaSO_4_, for which a decrease was found (*p* = 0.03) (see Figure 8d). Focusing on the shape of plasticization peak curves at the yield point, one can observe that the region becomes narrower and appears at lower strains as the BaSO_4_ content increases in composites (see Figure 8b). Moreover, yield plateau stress after plasticization peak decreased to lower stress levels, being significant for compositions of up to 15 wt.% BaSO_4_ (*p* = 0.04) (see Figure 8e). This drop in stress after the peak is indicative of cavitation induced by debonding of matrix–reinforcement interphase, and thus it might be concluded that filler content has a negative effect.

Regarding ductility, some decrease in elongation at break was observed at higher amounts of reinforcement in composites in regard to neat PCL. However, it is noticeable that all samples regardless of BaSO_4_ amount remained ductile with values of elongation at break higher than 557% (see Table 5 and Figure 8f). This result is statistically significant for 5 wt.% (*p* = 0.01), and the decreasing tendency for high content is notable (*p* = 0.065 for 35 wt.% BaSO_4_).

The results demonstrate that the BaSO_4_ particles in PCL are acting in the linear strain zone at low strains of the tensile stress–strain curve (see Figure 8a) as a usual stiff reinforcement for polymers. The increase in modulus in PCL with inorganic reinforcements is quite common [28,30,36,42]. The effect of the matrix-particle interfaces becomes relevant and affects the mechanical properties at higher strain values of the stress–strain curves. If the interface adhesion is poor [29], an increase in yield strength is not expected; debonding at the interfaces would prevent the reinforcing effect at the strain value at which the yield point appears. Interface debonding and cavitation explain the behavior of the yield region in which the strain is observed to appear at lower strain values; hence, yield stress drops for high amounts of BaSO_4_. This behavior is consistent with previous works provided for PCL composites, which evidenced a decrease in yield stress even when compatibilizing agents for the interface were used to improve the adhesion level [28]. Barium sulfate as reinforcement with PCL behaves similarly to calcium sulfates, which show increments in yield stress at medium reinforcement amounts and then a decrease in yield stress at >15 wt.% [32]. The behavior observed here for the PCL matrix also corroborates effects found for BaSO_4_ composites with other semicrystalline matrices such as polypropylene (PP) or polyethylene (PE) [23,43,44]. In this work, the falling in yield strength was not observed as BaSO_4_ particle content increased to a high amount (35 wt.%). This fact suggests a contributing effect of the crystal nucleation and growth in presence of particles, as also observed by DSC and PLOM, leading to an increased degree of crystallinity in PCL composites that compensates the detrimental effects of cavitation and debonding.

Finally, the impact of BaSO_4_ particles on the strain capacity of the polymer matrix can be discussed for submicron and nano-sized particles, as it has been proved that they provide an enhancement in the toughness of PLA at low particle amounts (≤10 wt.%) [21,44]. However, for higher particle amounts, a decrease in toughness is expected because debonding and cavitation occur, leading to a decrease in the strain capacity of composite formulations. Micron-sized holes, coalescence and, finally, cracks that develop during stretching of samples lead to decreasing values of elongation at break, as was observed here for PCL-BaSO_4_ composites. Therefore, it can be concluded that the addition of BaSO_4_ particles in quantities from 5 to 25 wt.% is optimal for polymer composites of PCL without a detrimental effect on toughness.

### 3.4. X-ray Imaging and Radiopacity Assessment

**Figure 9:**
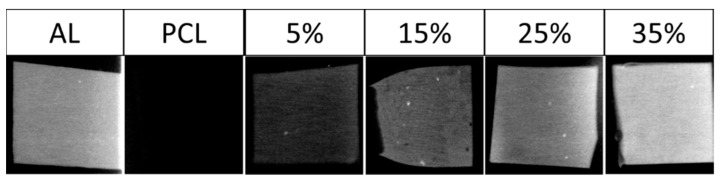
X-ray images of aluminum, PCL and PCL–barium sulfate composites.

Figure 9 shows the X-ray images of 1 mm thickness films of PCL and its 5, 15, 25 and 35 wt.% barium sulfate composites compared to 1 mm thickness aluminum film. Barium sulfate particles increased the radiopacity of PCL, as expected. Samples with 5 wt.% barium sulfate were detectable, and samples with 25 wt.% barium sulfate had radiopacity comparable to that of aluminum.

## 4. Conclusions

The main goal of the current study was to study the relationship between crystallization behavior, mechanical properties and radiopacity of poly(ε-caprolactone) (PCL) and its barium sulfate (BaSO_4_) composites. In this work, we prepared samples of PCL with different contents (0, 5, 15, 25 and 35 wt.%) of submicron particles of BaSO_4_ with an average size of 577 nm.

Thermal studies showed a statistically significant increase in PCL degree of crystallinity during cooling from melt in presence of BaSO_4_ particles. Non-isothermal crystallization kinetics of PCL in the absence and presence of BaSO_4_ particles were assessed at 1, 5 and 10 °C min^−1^ cooling rates from melt. The nucleating effect of BaSO_4_ was found to change the morphology and degree of crystallinity of the primary crystals of PCL, which was also corroborated by the use of a polarized light optical microscope (PLOM). The experimental data were well fitted to the Avrami-Ozawa-Jeziorny model, and the determination of the corresponding parameters revealed a secondary crystallization that contributes to an increase in the degree of crystallinity with internal structure reorganization.

The addition of barium sulfate particles in composite formulations with PCL improved stiffness, but the other relevant mechanical properties (strength, elongation and toughness) were not optimal. This is attributed to the debonding and cavitation at the particle–matrix interfaces in which adhesion plays a pivotal role.

Moreover, X-ray images show that 15 wt.% BaSO_4_ is sufficient to present radiopacity for devices to be visible for use in medical imaging techniques.

One of the more significant findings to emerge from this study is that PCL presents sufficient radiopacity with tough mechanical properties when mixed with 25 wt.% BaSO_4_ submicron particles. This finding is of broad use to the scientific and biomedical communities, as this composite fulfilled the required conditions for monitoring implants and drug delivery devices by X-ray imaging techniques.

## Figures and Tables

**Table 1:** Name codes of composite samples showing real barium sulfate content after processing as calculated by TGA analysis.

Sample (Name Code)	BaSO_4_ Content (wt.%)	Real BaSO_4_ Content (wt.%)
PCL	0	0
PCL_05BaSO_4_	5	8.4
PCL_15BaSO_4_	15	14.2
PCL_25BaSO_4_	25	25.0
PCL_35BaSO_4_	35	32.5

**Table 2:** Thermal properties and degree of crystallinity of poly(ε-caprolactone) (PCL) and its BaSO_4_ composites. *T*_m_: melting temperature; Δ*H*_m_: melting enthalpy; *X*_c_: degree of crystallinity.

Sample (Name Code)	BaSO_4_ Content (wt. %)	*T*_m_ (°C)	Δ*H*_m_ (J/g)	*X*_c_ (%)
PCL	0	67.0 ± 0.8	70.5 ± 1.1	46
PCL_05BaSO_4_	8.4	65.3 ± 0.8	68.2 ± 0.7	49
PCL_15BaSO_4_	14.2	64.5 ± 0.5	64.8 ± 0.6	49
PCL_25BaSO_4_	25.0	64.2 ± 0.5	59.1 ± 1.0	52
PCL_35BaSO_4_	32.5	63.4 ± 1.2	52.6 ± 2.0	51

**Table 3:** Crystallization temperature and crystallization enthalpy of PCL and its BaSO_4_ composites at different cooling rates.

Sample (Name Code)	Cooling Rate (°C min^−1^)	*T*_c_ (°C)	Δ*H*_c_ (J g^−1^)
PCL	1	33.7	62.3
	5	28.7	57.7
	10	24.8	54.1
PCL_05BaSO_4_	1	37.8	67.9
	5	32.8	62.6
	10	29.9	59.6
PCL_15BaSO_4_	1	37.5	68.1
	5	31.8	63.0
	10	28.8	60.4
PCL_25BaSO_4_	1	38.5	71.2
	5	33.2	66.5
	10	30.7	63.7

**Table 4:** The parameters of the double linear Avrami–Ozama–Jeziorny model for PCL and its BaSO_4_ composites at different cooling rates.

Sample (Name Code)	Φ (°C min^−1^)	*Z*_c1_ × 10^6^	*n*_1_	*r*_1_	*Z*_c2_ × 10^6^	*n*_2_	*r*_2_
PCL	1	393	3.06	0.96	112,010	1.12	0.90
	5	26,661	3.00	0.97	134,567	1.24	0.96
	10	93,981	2.46	0.97	138,174	1.65	0.98
PCL_05BaSO_4_	1	311	3.76	0.94	156,060	1.01	0.91
	5	22,345	3.68	0.94	157,177	1.08	0.95
	10	130,814	3.69	0.97	149,402	1.33	0.96
PCL_15BaSO_4_	1	353	3.68	0.91	171,962	0.98	0.92
	5	24,246	3.73	0.95	159,310	1.09	0.94
	10	145,824	3.65	0.97	161,103	1.30	0.95
PCL_25BaSO_4_	1	266	3.86	0.91	173,790	0.99	0.90
	5	25,408	3.79	0.95	169,341	1.05	0.94
	10	169,961	3.81	0.97	170,617	1.22	0.93
PCL_35BaSO_4_	1	339	3.70	0.91	136,477	1.08	0.89
	5	21,298	3.72	0.95	144,059	1.17	0.94
	10	229,399	3.92	0.97	147,983	1.39	0.92

**Table 5:** Values of the mechanical properties measured by tensile test for neat PCL and its BaSO_4_ composites. Young’s modulus (*E*), yield stress and yield strain (σ_y,_ ε_y_), yield plateau stress (σ_yp_) and elongation at break (ε_b_)

Sample (Name Code)	E* (MPa)	σ_y_ (MPa)	ε_y_ (%)	σ_yp_ (MPa)	ε_b_ (%)
PCL	308 ± 9	13.8 ± 0.5	10.2 ± 1.5	12.1 ± 0.2	714 ± 27
PCL_05BaSO_4_	336 ± 11	14.1 ± 0.3	10.9 ± 0.8	11.9 ± 0.2	841 ± 23
PCL_15BaSO_4_	353 ± 4	13.7 ± 0.2	9.9 ± 0.3	11.4 ± 0.2	615 ± 92
PCL_25BaSO_4_	368 ± 8	13.6 ± 0.3	8.8 ± 0.7	11.3 ± 0.2	623 ± 28
PCL_35BaSO_4_	397 ± 19	12.7 ± 0.2	6.5 ± 0.3	10.1 ± 0.1	557 ± 50

* E is defined by linear fitting at first 2% of strain.

## Data Availability

Data available on request due to restrictions eg privacy or ethical. The data presented in this study are available on request from the corresponding author. The data are not publicly available due to the private character of the research.
